# The Impact of Gastronomic Tourism on Thailand Economy: Under the Situation of COVID-19 Pandemic

**DOI:** 10.1177/21582440231154803

**Published:** 2023-02-20

**Authors:** Pairach Piboonrungroj, Satawat Wannapan, Chukiat Chaiboonsri

**Affiliations:** 1 Chiang Mai University, Thailand

**Keywords:** COVID-19 pandemic, gastronomic tourism, Thai’s economic impact, dynamic input-output analysis, Bayesian Structural Time Series (BSTS)

## Abstract

With the COVID-19 pandemic’s complexity and inexorable devastation, this research article attempts to forecast Thailand’s economic move forward through gastronomic tourism promotion. The dynamic input-output (I-O) model was the primary method for classifying gastronomic activities in tourism I-O data, which was investigated sector by sector. The Ministry of Tourism and Sports in Bangkok, Thailand, officially gathered the 2017 I-O table. To briefly explain the empirical results, it found that the main sectors of gastronomic tourism that highly impact Thailand’s economy are the processing and preserving of foods, other foods, food and beverage serving activities, and other food services. In terms of forecasting during the period of the COVID-19 pandemic, the Bayesian Structural Time Series (BSTS) based on the dynamic input-output (I-O) model suggests that approximately 1% to 2% of Thailand’s gastronomic tourism will be able to contribute to the GDP of this country substantially. By the way, if this research result is significant, then both the private sector and the government sector need to be concerned and promote those sectors as much as they can.

## Introduction

**Figure 1.**
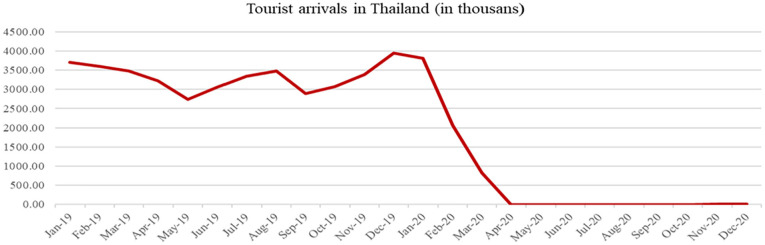
Historical data of tourist arrivals in Thailand. *Source.* Bank of Thailand.

A historical story Gastronomic tourism can be traced back to an ancient period of Greek culture. The word “gastro” refers to the stomach, and “nomos” means knowledge (Scarpato, 2002). Many researchers around the world have already confirmed that gastronomic tourism has a high potential being a new trend tourism movement activity to support tourism industry development sustainably (Citrinot, 2018; Kivela & Crotts, 2006; Lunchaprasith, 2018; Promnil et al., 2021; Rimdusit & Duangsaeng, 2019). Suanpang (2015) also proposed that gastronomic tourism is a high-potential new trend in tourism activity in ASEAN. Unfortunately, it is nearly the end of 2019, which this year has started to signal a negative impact on tourism around the world. The situation in COVID-19 is able to disrupt not only economic growth but also the tourism sector as well. This pandemic is the key player in bringing down the tourism sector around the world, especially in Thailand. The chronic depression in World economic activities caused by the COVID-19 pandemic is still unstoppable (Mohsin et al., 2021). The locked-down policy is simple to launch, but it is not for long. Thailand is one of the countries shutting down major infectious cities (Dechsupa et al., 2020). Unfortunately, most cities are highlighted in tourism areas such as Bangkok, Phuket, even Chiang Mai. The policy directly damages tourism activities and stops the number of tourist arrivals. This fact is graphically shown in Figure 1. The number of tourists inbounds is suddenly fallen near-zero people during the second quarter in 2020. The unfavorable trend is chronical in the whole year. To cure and revive tourism sectors in a short term is nearly impossible. The major query for this issue is what activity can refresh both domestic and foreign tourists and persuade them to travel with confidence.

Gastronomic tourism has been highly mentioned as an “emerging tourism market” in many countries around the world from 2014 until the present time (Berbel-Pineda et al., 2019; Dancausa Millán, 2021; Diaconescu et al., 2016; Gheorghe et al., 2014; Guruge, 2020; Hernández-Rojas et al., 2021; Hernández-Rojas & Huete Alcocer, 2021) In terms of traveling for food, this represents a hunger tourist seeking uniqueness, culture, preparation processes or production, and eating manners, and it is also a part of creative tourism (Boonpienpon & Wongwiwattana, 2017). In Thailand, gastronomic tourism is now being considered a novel tool for reviving the great depression of the tourism sector, especially during COVID-19 (Kattiyapornpong et al., 2021).

**Figure 2.**
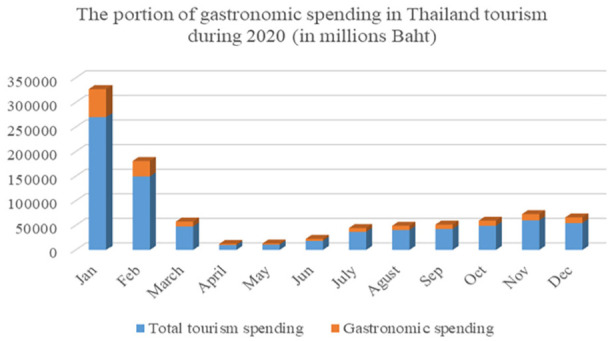
The portion of gastronomic tourism in terms of tourist spending. *Source.* Ministry of Tourism and Sports, Bangkok, Thailand.

**Figure 3.**
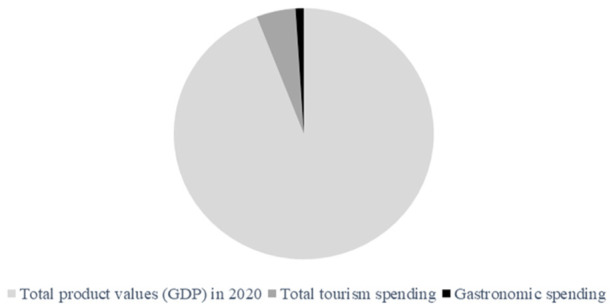
The pie chart presents a visualization of gastronomic activities in the whole production value of Thailand. The total tourism spending is in one of ten of the total product values (GDP). Gastronomic tourism spending is 20% of the total tourism spending. *Source.* Ministry of Tourism and Sports, Bangkok, Thailand.

As shown in Figures 2 and 3, approximately 20% of total tourism spending in Thailand is based on the assumption that the pandemic can be systematically controlled by 2020. It is reasonable to assert that gastronomic tourism should already be one alternative key driving tourism in the future. Unfortunately, there are only a few researchers who attempt to spotlight gastronomic tourism in Thailand, particularly during and post the COVID-19 pandemic. Furthermore, Suanpang (2015) also found that Thailand affirms it can be “the hub of gastronomic tourism in ASEAN” as soon as possible. Moreover, Kattiyapornpong et al. (2021) stated that gastronomic tourism, particularly gourmet experiences, is vital to driving visitor spending during the COVID-19 pandemic via platforms that offer virtual gastronomic tourism activities. Based on data confirmation again, Figure 3 visualizes the pie chart and confirms that the role of gastronomic activities has the potential to drive some sectors of the Thai tourism industry forward in the future, especially during and after the COVID-19 pandemic. The research gaps in gastronomic tourism in Thailand continue to be a milestone challenge for researchers. Once again, the aim of this research article attempts to emphasize how foresight in the future how to maintain and stimulate the Thai economy during and after the COVID-19 pandemic. In order to fulfill the fulfillment of answering the research gaps, this research article was organized into the following four parts: Part 1 displays a literature review on the scope of the tourism industry and gastronomic tourism. Part 2, which demonstrates the scope of the study, and Part 3, which includes the research methodology that was applied in the process of the research study. The final part is a part of the discussion and conclusion of the research study.

## Literature Review

### Is It Time for Thailand Tourism to Make a Reconsideration?

As a historical trend of international tourists arriving in Thailand territory seemed fantastic, a traditional perspective of tourism improvement hangs on quantities, not quality. As stated by Wannapan and Chaiboonsri (2017), the number of Chinese tourists who came to Thailand for traveling provided enthusiastic activities only for a short term. The demand side is seemed to be intensively mentioned by authorities rather than looking back to the supply side. The huge problem is how to maintain tourism viability when uncertainties suddenly occur in terms of intensive demand promotions. Before the Coronavirus pandemic shuts down the heart of tourism in Thailand, numbers of inbound tourists are the index to predictive monitor tourism viability such as Song et al. (2003) and Unthong et al. (2015). Apart from only focusing on the number of arrivals, the factor of international trades was issued to be an influence improved the tourism demand in Thailand (Chaisumpunsakul & Pholphirul, 2018). This indicator referred to the rate of international openness, which was a direct effect on the types of meeting tourism, conference tourism, even exhibition tourism. Additionally, stated by Preedatham et al. (2018), domestic prices in markets and safety travels are concluded to be factors improving tourism demands in Thailand’s tourism sectors. Tran et al. (2020) also concluded the best solution for stimulating tourism sectors is safety, prevention, and control. As literature, however, it seems a highlight of gastronomy is still overlooked by empirically econometric studies in Thailand. Most of Thai gastronomic tourism researches only focused on specific areas, not gastronomy industries, such as Bunrangsee et al. (2018) reviewed the gastronomic tourism model applying for the Thailand gastronomic tourism with literature from successful food tourism countries (e.g., France, Hong Kong, Republic of Korea, and Japan). Srihirun and Sawant (2019) studied the plans and policies of DASTA and TAT on creative tourism in Bangkok cities using content analysis. Tunming et al. (2020) explored specific areas in the Greater Mekong Sub-Region (GMS) for promoting the gastronomic tourism of Tai-Dam’s ethnic indigenous identities. As a consequence, this is a good chance gastronomic Tourism is raised to be the talk of the town in terms of the solution for Thai tourism.

### Can a Sectorial Tourism Overview Be a Solution?

It is obvious that the Coronavirus pandemic causes the fallen of tourism demands. To pump up the number of international tourists is a difficult task and this downsizing impact is not soon backed to a normal situation. The huge gap of researches is an exploration of down and upper streams in tourism sectors. The study on an econometric model and a computable general equilibrium model for the tourism structure affecting the outputs of overcapacity industries is highlighted. Li and Song (2013) started to evaluate the impacts of large-scale events on tourism demand using the general equilibrium computation (CGE model). The visa restriction was selected to be an example that a restricted measurement for tourism activities generated economic losses. Li et al. (2019) contributed the manuscript to investigate the impact of international tourists arriving in China on reform in provinces with severer industry overcapacities. The research found the crucial fact that the number of arrivals is a strong structural link to industrial viability widely. In 2020, the general equilibrium view of Thai tourism was explored by Wickramasinghe and Naranpanawa (2022). This article primarily reviewed CGE applications in tourism over the past 25 years using a systematic quantitative literature review approach. For ASEAN studies, starting at the basic model of input-output computations, a few academic papers studied the sectorial analysis based on an input-output (I-O) table. Although the I-O table of Thailand is widely recognized by many researchers such as Papong et al. (2015), Kuroiwa (2016), and Chaivichayachat (2020), a sectorial tourism input-output analysis still has none. Hence, using the I-O table to analyze down and upper streams in tourism sectors can be a novel chance for systematically understanding the root of this chronic tourism depression and efficiently providing a suitable way out of this problem, especially the urgent optimal choice for Thailand tourism refreshment.

### Gastronomic Tourism is Significant and Potential to Drive the Tourism Industry

Recently, gastronomic tourism has played an important role in driving the tourism industry in many aspects around the world. It is very substantial for stimulating the tourism industry and can be found in much tourism research. For example, Dancausa Millán et al. (2021) studied the demand for gastronomic tourism in Andalusia (Spain). This research result found that gastronomic tourism plays a significant role in attracting a greater number of tourists arriving in Andalusia (Spain) and can also generate more income for Andalusia’s economy as well. Hernández-Rojas et al. (2021) investigated the impact of restaurant branding and gastronomy on tourist loyalty in Córdoba, Spain. They found that gastronomic tourism has become a fundamental pillar to stimulate the tourism industry in Córdoba (Spain) to move forward. Moreover, Hernández-Rojas et al. (2021) studied the role of the traditional restaurant in impacting tourist arrivals in Córdoba, which is a city in southern Spain. The empirical results of the research found that satisfaction with traditional restaurants has a significant positive effect on attracting tourists to revisit this city. Furthermore, it can generate income for this city, which is driven by gastronomic tourism in substantial amounts.

Based on all the results of gastronomic tourism research, it can be affirmed that traveling food tourism is an important activity that has potential stimulation for economic development based on a tourism-oriented economy.

## The Scope of Objective and Data Review

**Figure 4.**
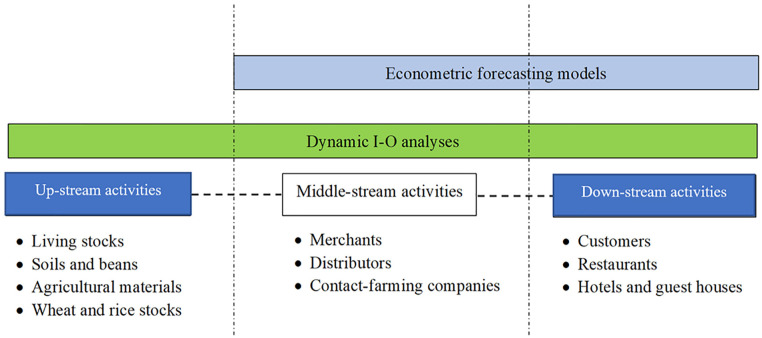
The scope of research.

At the moment this article is conducted, negative impacts caused by the pandemic continuously destruct tourism activities and their sectorial relevancies. The main aim to academically explore sensible solutions for saving tourism in Thailand is mentioned as a “priority.” Gastronomic traveling is the role framework for addressing this difficult situation. The details displayed in Figure 4 explain the conceptual framework of this study. In other words, the whole stream (upstream to downstream of tourism activities) is scoped by two econometric models. The first method—“Bayesian structural time-series”—is a predictive scenario employed to point out initial signs for the next 3 years (2021–2023). This section covers the activities between midstream and upstream. The second method is also a predictively sectorial analysis.

This method—“Dynamic I-O analysis”—is employed to complete the explanation of tourism productive lines. The whole stream is computationally merged. Macroeconomically, the economic improvement by gastronomic tourism is then classified.

### Data for the Sectoral Tourism Analysis

Because gastronomic activities widely link to other sectorial production lines, a traditional assumption to fix constants for non-considering sectors (referring to as exogenous variables) is suspicious. For this process, 2017 Thailand tourism input-output data was observed from the Ministry of Tourism and Sports. Because the I-O table is updated every 5 years, the next one will be approximately launched during 2023. This is a reason that the data is the most updated table for the I-O analysis. Sectorial details (89 sampling sectors) of Thailand’s tourism activities are demonstrated in Table 1.

**Table 1.** Details of Thailand Input-Output Table Updated Till 2017.

Codes	Sector details
001	Paddy stockpiles
002	Maize stockpiles
003	Cassava stockpiles
004	Bean and nut stockpiles
005	Vegetable and fruit stockpiles
006	Sugarcanes
007	Rubber (Latex)
008	Other Crops
009	Livestock
010	Forestry
011	Fishery
012	Crude oil and coal
013	Metal ore
014	Non-metal ore
015	Abattoir
016	Processing and preserving of foods
017	Rice and other grain milling
018	Sugar refineries
019	Other foods
020	Animal foods
021	Beverages
022	Tobacco processing and products
023	Spinning, weaving and bleaching
024	Textile products
025	Papers and paper products
026	Printing and publishing
027	Basic chemical products
028	Fertilizer and pesticides
029	Other chemical products
030	Petroleum refineries
031	Rubber products
032	Plastic wares
033	Cement and concrete products
034	Other non-metallic products
035	Iron and steel
036	Non-ferrous metal
037	Fabricated metal products
038	Industrial machinery
039	Electrical machinery and apparatus
040	Motor vehicles and repairing
041	Other transportation equipment
042	Leather products
043	Saw mills and wood products
044	Other manufacturing products
045	Electricity and gas
046	Water works and supply
047	Building construction
048	Public works and other construction
049	Specific retails of specific tourism countries containing characteristic goods
050	Wholesale and retail trades
051	Hotels and resorts
052	Guesthouses
053	Home stays/community based tourism/rural tourism
054	Food and beverage serving activities
055	Other food services
056	Drinking places
057	Other beverage services
058	Interurban and rural bus transportations
059	Passenger bus and other local transportations
060	Nonscheduled transit passenger transportations
061	Road passenger transports
062	Railway’s passenger transports
063	Water passenger transports
064	Air passenger transports
065	Transport equipment rentals
066	Travel agencies and other reservation services activities
067	Other transports
068	Communication
069	Banking and insurance
070	Real estates
071	Business services
072	Public services
073	Performing arts and nature reserve services
074	Museum and preservation services
075	Botanical and zoological garden services
076	Independent artists
077	Spa and massages
078	Golf courses
079	Adventure travels and extreme sports
080	Amusement parks and theme parks
081	Recreational activities and entertainment
082	Participant sport
083	Conference centers and exhibition
084	Other vehicle rentals
085	Health care services
086	Service training/service training of culture/recovery language school
087	personal service for tourism
088	Other services
089	Unclassified

*Source.* Ministry of Tourism and Sports, Bangkok, Thailand.

## Methodology and Empirical Application

### Bayesian Structural Time Series (BSTS) for the Predictive Impacts of COVID-19

As time passes, the main aim of this paper does take account into a predictive visualization of the Thai economic system walking through the pandemic from 2021 to 2023. The predicted trends were in the scenario specifically assumed Thai GDP was fluctuated by monthly gastronomic tourism spending in 2020. Bayesian structural time series (BSTS) is a powerful computational model used to analyze the keyword data over time. The power of Bayesian statistics is broadly applied in various types of researches such as regression, classification, clustering, and time series forecasting (Jun, 2019; Ross, 2012). Based on the Bayes theorem, a structure of Bayesian statistics is expressed as follows:

(1) $$P\left(\theta |X\right)=\;\frac{P\left(P\left(X|\theta \right)\right)}{P\left(X\right)},$$

where *X* is an observed sample (raw data) and θ is a hyperparameter (updated data). P(X) is defined as follows: $P\left(X\right)=\;\sum_{\theta }P\left(X,\theta \right)=\;\sum_{\theta }P\left(X|\theta \right)P\left(\theta \right)$. P(θ) stands for the model prior which represents the character of “subjective thinking,” and Gaussian distribution based on the occurred frequency values from 0 to infinite [0,∞]. is selected to be the prior. Also, P(X|θ) represents the likelihood function combining with a simulation method, namely “Markov Chain Monte Carlo: MCMC” (Metropolis et al., 1953). This tool empowers Bayesian statistics through the process of updated random information by learning the observed data *X* given θ (likelihood). Consequently, the P(θ|X) as posterior of the random parameter. The basic structure of the BSTS model is the following equation (Jun, 2019):

(2) $$y_{t}=\;\mu_{t}+\;\tau_{t}+\;\beta^{T}X_{t}+\;\varepsilon_{t},$$

where $\mu_{t}=\;\mu_{t-1}+\;\delta_{t-1}+\;\mu_{t}$, $\delta_{t}=\;\delta_{t-1}+\;v_{t}$, and $\tau_{t}=-\sum_{s=s}^{S-1}\tau_{t-s}+\;\omega_{t}$. The $\tau_{t}$ is a Gaussian distribution. Hence, the Bayesian regression model is derived as follows (Gelman et al., 2013; Jun, 2019):

(3) $$y=\;\beta_{0}+\;\sum_{i=1}^{p}\beta_{i}X_{i}+\;e,$$

Where e is the Gaussian distribution containing mean = 0 and variance = $\sigma^{2}$. In terms of forecasting scenarios representing in this paper, the prior (initial value) of $\sigma^{2}$ is relied on an inverse-chi square $\left(\mathrm{Inv}-\chi^{2}\right)$ distribution as follows (Gelman et al., 2013; Jun, 2019):

(4) $$\sigma^{2}\;\sim \;\mathrm{Inv}-\;\chi^{2}\;\left(n-p,s^{2}\right)$$

Where *n* and *p* are updated information and parameter sizes. To compute $s^{2}$ is demonstrated in the following equation (Jun, 2019):

(5) $$s^{2}=\;\frac{1}{n-p}{\left(y-X\hat{\beta }\right)}^{T}\left(y-X\hat{\beta }\right)$$

**Figure A1.**
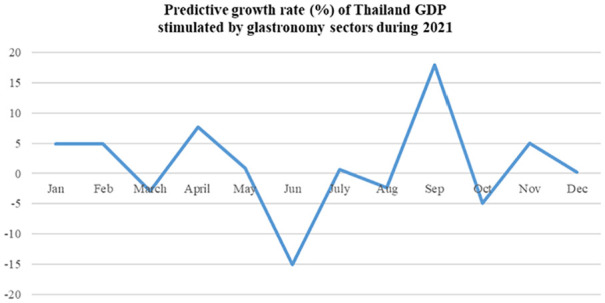
The monthly predictive trend of GDP driven by gastronomic activities.

**Figure A2.**
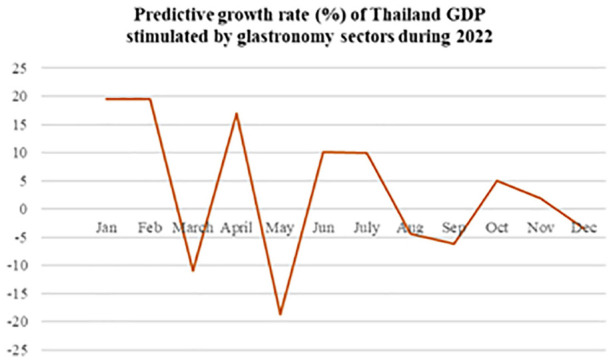
The monthly predictive trend of GDP driven by gastronomic activities.

**Figure A3.**
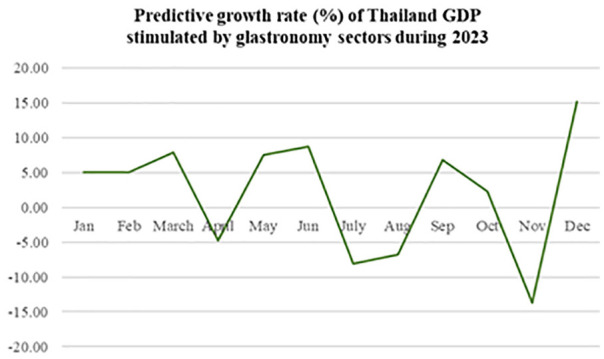
The monthly predictive trend of GDP driven by gastronomic activities.

To address an empirical application, the BSTS model was employed to predict expansion rates of Thailand GDP systematically driven by gastronomic tourism (spending for foods and beverages) from 2021 to 2023. As stated by Srichannil (2020), Thailand appeared to be medically well prepared to address the panic infectious disease. Based on the prediction, three positive scenarios for 3-year predictions were presented in Table A1 (see Appendix A). The predictive results of gastronomic tourism contributing to Thailand’s economy were 1.07, 1.79, and 1.86 per year (the Bayesian structural time series [BSTS] prediction result), respectively. The prediction was more detailed graphically as monthly trends by Figures A1–A3 (see Appendix A). These indicators would be used to be initial impacts on the dynamic sectorial analysis based on I-O information. More importantly, underneath the unstoppable outbreak of the COVID-19 pandemic, the paper’s scenario assumed to revive tourism in Thailand from 2021 to 2023 by domestic gastronomic tourism. Under this scenario, although the number of foreign tourists was the critical factor strongly directly impacts on tourism industries (Li et al., 2019), international tourists were not accounted as the factor which potentially drive the tourism refreshment because of its dramatic decreasing (near zero person). Consequently, the predictive 3-year of Thailand’s gastronomic contribution to GDP in terms of income distribution indicated a positive-looking scenario by focusing on internal systematic development.

### Dynamic I-O Analysis for Clarifying the Driven Impact of Gastronomic Tourism on 89 Sectors in the Tourism I-O Table of Thailand

The dynamic I-O model was developed to deal with the restriction and it was improved to be available for a scenario in which ratios of input factors fluctuated over time. In other words, *a_ij_* refers to the technical coefficient of good (*i*) need for one unit of production of good (*j*). The value of *a_ij_* is supposed to be fully used for calculation of each economic sector or each of industry sector in economy. However, it is not always representing the real situation for analysis in economy. For the dynamic model, the *b_ij_* refer to capital coefficient of outputs from production sector (*i*) and the economic sector (*j*) need to accumulate for capital stock, equals *v_ij_* divide by *x_ij_* which it can be simplified as

(6) $$b_{\mathrm{ij}}=\;\frac{v_{\mathrm{ij}}}{x_{j}}.$$

Once again, the *b_ij_* is the capital coefficient need to add into the original IO model (static IO model). Which it can more accurately or reliably by revision of the old version for the IO model to be the new version of IO model as the dynamic IO model. In a general way, the dynamic I-O model can be written by a mathematical formula (see Equation 7)

(7) $$x_{i}^{t}=\;\sum_{j=1}^{n}a_{\mathrm{ij}}x_{j}^{t}+\;\sum_{j=1}^{n}b_{\mathrm{ij}}\left(x_{j}^{t+1}-x_{j}^{t}\right)+\;y_{i}^{t}$$

In the first terms of Equation 7, it means that the outputs of all production sectors *i* ($x_{i}^{t}$) were carrying outed to be the input factor in the process of production for all economic sector (89 sectors) or industry sector in the economy (See Equation 8).

(8) $$\sum_{j=1}^{n}a_{\mathrm{ij}}x_{j}^{t}$$

In the second term of Equation 7 was represented by the time changing for a new output (*i*) was employed to be the capital input to produce the output (*j*) of economic sectors in the economy. Because of the time changing impact on every economic sector (Šafr, 2016) especially 89 sectors in Thailand (Chen et al., 2021), which it is can be written the mathematical formula by Equation 9

(9) $$\sum_{j=1}^{n}b_{\mathrm{ij}}\left(x_{j}^{t+1}-x_{j}^{t}\right),$$

Therefore, the final output of every economic sector, which they are equal to $y_{i}^{t}$ as presented below

(10.1) $$x_{i}^{t}=\;\sum_{j=1}^{n}a_{\mathrm{ij}}x_{j}^{t}+\;\sum_{j=1}^{n}b_{\mathrm{ij}}\left(x_{j}^{t+1}-x_{j}^{t}\right)+\;y_{i}^{t},$$

(10.2) $$y_{i}^{t}=\;x_{i}^{t}-\;\sum_{j=1}^{n}a_{\mathrm{ij}}x_{j}^{t}+\;\sum_{j=1}^{n}b_{\mathrm{ij}}\left(x_{j}^{t+1}-x_{j}^{t}\right),$$

(10.3) $$y_{i}^{t}=\;x_{i}^{t}-\;\sum_{j=1}^{n}a_{\mathrm{ij}}x_{j}^{t}+\;\sum_{j=1}^{n}b_{\mathrm{ij}}x_{j}^{t}-\;\sum_{j=1}^{n}b_{\mathrm{ij}}x_{j}^{t+1}$$

For computation of full Dynamic IO model for analysis the impact of gastronomic tourism on Thailand economy under the situation of COVID-19 disruption would be utilized by Equation 11 as displayed below.

(11) $$Y^{t}=\;\left(I-A+B\right)X^{t}-BX^{t+1}$$

Where

*I*: The Identity Matrix (*n* × *n*) as the matrix of 89 economic sector of Thailand economy

*A*: The Input Coefficient Matrix (*n* × *n*) as the matrix of 89 economic sector of Thailand economy

*B*: The Capital Coefficient Matrix (*n* × *n*) as the matrix of 89 economic sector of Thailand economy

*X*^*t*^: The Output Matrix at time *t* (*n* × *n*), *t* = 0, 1, … , *T* as the output of 89 economic sector of Thailand economy

*X*^*t*+1^: The output matrix at time *t* + 1 (*n* × *n*), *t* = 0, 1, … , *T* as the output of 89 economic sector of Thailand economy

*Y*^*t*^: The final demand matrix at time *t* (*n* × *n*), *t* = 0, 1, … , *T* as the final demand of 89 economic sector of Thailand economy

For the application using to ultimately provide dynamic predictions of sectorial down and upper streams in Thailand tourism, gastronomic activities were empirically a driven factor systematically refreshing backward and forward productive lines.

Along 89 sections inside the collected tourism I-O table, productive activities which were in the scope of gastronomy were represented in Table 2. The result is clear that gastronomic tourism deeply links with agricultural activities, especially full-stream production lines such as livestock, preserving foods, rice and grain milling stockpiles. These activities drove the development of rural and food tourism with a double rate of tourism multipliers (larger than one). In other words, gastronomical heritages (i.e., regional food, organic food) can be incorporated in rural small enterprises, agricultural employment, and domestic tourism (Sidali et al., 2011). Although gastronomic activities are obvious the tool for systematically refreshing tourism in Thailand, there are several issues that detail sectors need to be reconsidered a deadweight loss. The upstream sector (FLI > 1 and BLI > 1) (both the supply-push and the demand-pull (Gorska, 2015) and the midstream sector (FLI < 1 and BLI > 1) (only the demand-pull) consist of the processing and preserving of foods, other foods, food and beverage serving activities, and other food services (see more details in Table 2).Another issue that was important for the downstream production line sector in the tourism industry of Thailand would be the vegetables and fruits, beverages, drinking places, and other beverage services that need to be considered as well (see detail in Table 2). However, their multipliers were still more than one, meaning that these sectors still played an important role in stimulating Thailand’s economy, especially through gastronomic tourism. Nevertheless, they did not provide benefits for the whole production line in Thailand’s tourism (FLI < 1 and BLI < 1)

**Table 2.** The Predominant Gastronomic Outputs of Thailand Tourism System During.

Tourism activities	Multiplier	Backward linkage	Forward linkage	FLI > 1	FLI < 1	FLI < 1
				BLI > 1	BLI > 1	BLI < 1
Vegetables and fruits	1.779	0.781	0.971	—	—	Yes
Processing and preserving of foods	2.748	1.206	1.171	Yes	—	—
Other food	2.533	1.112	0.664	—	Yes	—
Beverages	2.231	0.979	0.898	—		Yes
Food and beverage serving activities	2.701	1.185	0.624	—	Yes	—
Other food services	2.501	1.097	0.439	—	Yes	
Drinking places	2.160	0.948	0.498	—	—	Yes
Other beverage services	1.900	0.835	0.439	—	—	Yes

*Source.* Author’s computed.

Increase gastronomic tourism as a solution to rebooting Thailand’s macroeconomic tourism. The purpose of this research article is to use a dynamic I-O and the Bayesian structural time series (BSTS) model for both stimulation and prediction of Thailand’s economy under the COVID-19 pandemic.

However, from the standpoint of stimulation scenarios, Table 3 clearly shows a dynamic I-O model. Moreover, the prediction scenario of GDP growth that has been contributed by gastronomic tourism is confirmed by the Bayesian structural time series (BSTS) model, which is displayed in Table 3 as well. Table 3 shows the dynamic sectorial forecasting of Thailand’s economy driven by gastronomic tourism between 2021 and 2023. From Table 3, the vegetables and fruits were predicted to increase over the next 3 years, with the value of this sector from 120,974,520 baht to 213,683,970 baht. The processing and preserving of foods will contribute to Thailand’s economy increasing every year from 333,321.070 baht to 588,763,380 baht. Moreover, the other food from gastronomic tourism can contribute to the income of the GDP of this country for approximately 3 years from 185,877,350 baht to 328,325,420 baht. The beverages sector can be generated as income to the GDP of Thailand for 3 years, approximately from 547,816,380.00 baht to 967,638,290.00 baht.

**Table 3.** The Predictive Gastronomic Activities Driving Thailand’s Economy From 2021 to 2023 (Unit: Thousand Baht).

Sectors	Predictive economic values		
	2021	2022	2023
	1.07%	1.79%	1.86%
Vegetables and fruits	120,974.52	2,023,777.94	213,683.97
Processing and preserving of foods	333,321.07	557,611.88	588,763.38
Other food	185,877.35	310,953.70	328,325.42
Beverages	547,816.38	916,440.49	967,638.29
Food and beverage serving activities	8,613,414.74	14,409,357.32	15,214,349.35
Other food services	849,344.50	1,420,865.28	15,000,243.23
Drinking places	1,033,811.50	1,729,460.36	1,826,078.26
Other beverage services	331,147.65	553,975.98	584,924.39

*Source.* Author’s computed.

The top one in gastronomic tourism, which can be a high contributor of income to Thailand’s economy, especially during the period of the COVID-19 pandemic, is the food and beverage services activities. This sector can generate income for the GDP of Thailand from 2021 to 2023, which is 8,613,414,740 baht to 15,214,349,350 baht.

The other food services in gastronomic tourism can generate income for Thailand’s economy during the period of 2021 to 2023, approximately 849,344,500 baht to 15,000,243,230 baht. The drinking places may not be directly involved in gastronomic tourism; however, it is important for tourists to visit and consume the beverage, so it is included to generate income for Thailand’s economy as well. This sector will generate income for this economy of approximately 1,033,811,500 bahts up to 1,826,078,260 bahts during a period of 3 years (2021–2023). And the last sector in the gastronomic tourism of Thailand is beverage services, through which it can inject money into the Thai economy of around 331,147,650 baht in 2021 and add a value of up to 584,924,390 baht in 2023.

## Discussion and Recommendation

However, the situation of the tourism industry around the world is still uncertain, especially in Thailand. Twenty years ago, Thailand’s tourism industry played a more significant role in generating income for this country than 20% of its GDP. In 2019, foreign tourists arrive in Thailand in sufficient numbers to generate 2 trillion Baht (Surawattananon et al., 2021). This value can generate income for the GDP of Thailand by around 11% and can create jobs for the labor force of around 7 million people, or approximately 20% of total employment in Thailand. Unfortunately, in 2020, this sector will again encounter the situation of uncertainty caused by the COVID-19 pandemic (*www.statista.com*). From this situation, Thailand suffered a lot of problems in many dimensions. Especially with the reducing share of Thailand’s GDP from this sector. This year, Thailand’s economy received income from the tourism industry only around 6.78% of GDP. Consequently, gastronomic tourism may hopefully be one activity to stimulate this sector again, and hopefully, it will be in the spotlight in Thailand as soon as well.

It is common to state gastronomic activities are deeply blended with Thai societies. However, mass transportations, low-cost tourism promotions, even environmental careless tourism have been the role of tourism inspiration in several years ago. In last 10 years, the huge number of Chinese outbound has shaken Thailand tourism to be an extreme blooming. Unfortunately, stated by Wannapan and Chaiboonsri (2017), every party has to face an ending. Chinese tourists are no longer to be the key role of Thailand tourism. With stunning impacts caused by the COVID-19 pandemic, it seems no way out of this problem to revival tourism sectors in the country. A solution to refresh Thailand tourism comes from deeply looking back to domestic travels. This research article was one of pilot projects to promote “gastronomic activities.” The positive signs hinted at by Bayesian structural predictions and dynamic input-output forecasting confirmed that gastronomic tourism still played a significant role in stimulating the Thai economy, especially during and post the COVID-19 pandemic. Thai tourism can survive by foods and beverages. With plenty materials to be the center of gastronomic tourism in Asia, the flexible and efficient control for virus infections needs to be carefully mentioned. Particularly, tourism areas such as Northern and Southern regions should be politically relaxed, but they need to be intensively assured by efficient communications. Effective real information officially informed to the public is also a powerful tool to enhance gastronomic tourism with safety and cleanness and give a confidence to culinary businesses and industries in Thailand. Ultimately, the final result to sustainably implement gastronomic tourism was not only subsidizations from fiscal frameworks to people’s purchase power, but the modification of regulations, which allowed small medium enterprises (SMEs) to flexibly and simply access funds and opportunities, was inevitable because SMEs could more effectively deal with culinary activities in rural and urban areas than promoting mass tourism industries. This idea was supported by Rachão et al. (2018) concluded a deepen study on local food suppliers’ network linkages with two different approaches were needed: one in which the focus should be on independent medium-sized restaurants inside hotel chains and a second approach, regarding micro-scale restaurants (especially, those which were managed on a family business).

## Conclusion

The objectives to predictively provide scenarios underneath the chronic COVID-19 pandemic and dynamically demonstrated a sectorial development of gastronomic tourism were empirically analyzed in this paper. Experimentally based on the pandemic can be effectively controllable observed from monthly GDP data in 2020, the predictive scenarios were clear that gastronomic activities could be one of predominant factors driving positive impacts on Thai economy during next 3 years (2021–2023). With the flexible ability to explain random situations of Bayesian statistics combined with Markov Chain Monte Carlo (MCMC) simulations, the structural time-series model could give reasonable predictions along the upcoming 3 years, even though raw data was not large enough for a traditional asymptotic assumption. Thus, this section indicated that Thai economy could survive in a short-run. It was slightly recovered but it was not assured to sustainably expand. The huge gap to answer the query of gastronomic tourism being a sustainable component driving Thailand GDP was explored by applying dynamic input-output analyses for systematically monitoring gastronomic sectors in the official tourism I-O table provided by the Ministry of Tourism and Sports. The result was empirically clear that gastronomic activities could impressively improve economic values and tourism multipliers. Both down and upper streams of gastronomic industries could gain benefits. Moreover, this refers to significant development, particularly gastronomic tourism, as an important sector in the development of the tourism industry worldwide (Berbel-Pineda et al., 2019; Dancausa Millán, 2021; Hernández-Rojas et al., 2021; Hernández-Rojas & Huete Alcocer, 2021). For Thailand, gastronomic tourism is very challenging to understand. How it can be promoted and supported as a supplement to tourism, especially during and post the COVID-19 pandemic period. Many researchers in Thailand attempted to study for confirmation that gastronomic tourism needs to be promoted and supported more than now, such as Kivela and Crotts (2006), Citrinot (2018), Lunchaprasith (2018), and Promnil et al. (2021). Furthermore, Kattiyapornpong et al. (2021) proposed that during the COVID-19 pandemic, gastronomic tourism, particularly gastronomic experiences, is important to drive tourist spending via platforms offering virtual gastronomic tourism activity.

This research article still confirms that gastronomic tourism is an important activity to be supplementally to stimulate the tourism industry of Thailand, starting from upstream to midstream and finally downstream respectively. The research results concluded that the main sectors of gastronomic tourism that highly impact Thailand’s economy are the processing and preserving of foods, other foods, food and beverage serving activities, and other food services. In terms of forecasting during the period of the COVID-19 pandemic, the Bayesian Structural Time Series (BSTS) based on the dynamic input-output (I-O) model suggests that approximately 1% to 2% of Thailand’s gastronomic tourism will be able to contribute to the GDP of this country substantially. Remarkably, based on this methodology to forecast the GDP growth rate of Thailand, the gastronomic tourism-driven between 2022 and 2023 seems to be decreasing (see Table 3). This is a signal for both the private sector and the government sector that they need to be concerned and promote those sectors substantially to maintain a driven system to move on. Unfortunately, with the fact that the pandemic still damaged the world economy and it was not immediately stopped, gastronomic tourism may hopefully be the solution to bring Thailand’s domestic economy to be able to move forward in the next 3 years.

The future research from this research study will be limited because this research study still focuses on the macro level of gastronomic tourism based on the dynamics of the IO table of the tourism sector in Thailand. Therefore, future research needs to update the data in the dynamics IO table because the data on economic structure is changing very fast. If the researcher is able to find the data in the dynamics IO table, especially the updated IO for the tourism sector, then the result of the research may be more accurate in supporting and promoting gastronomic tourism to drive the economy significantly next.

## Appendix A

**Table A1.** Predictive Scenarios for Thailand’s GDP Growth Rates Driven by Domestic Gastronomy 2021 to 2023.

Time	Average predictive growth rate (%) in 2021	Average predictive growth rate (%) in 2022	Average predictive growth rate (%) in 2023
Average	1.07	1.79	1.86

*Source.* Authors’ calculations.
